# The effect of directional inertias added to pelvis and ankle on gait

**DOI:** 10.1186/1743-0003-10-40

**Published:** 2013-04-19

**Authors:** Jos H Meuleman, Edwin HF van Asseldonk, Herman van der Kooij

**Affiliations:** 1 Moog Robotics, Nieuw-Vennep, The Netherlands; 2 Department of Biomechanical Engineering, University of Twente, Enschede, The Netherlands; 3 Biomechanical Engineering, Delft University of Technology, Delft, The Netherlands

**Keywords:** Inertia, Kinematics, Pelvis, Metabolic rate, Locomotion, Leg loading, Emg, Robotic gait trainers

## Abstract

**Background:**

Gait training robots should display a minimum added inertia in order to allow normal walking. The effect of inertias in specific directions is yet unknown. We set up two experiments to assess the effect of inertia in anteroposterior (AP) direction to the ankle and AP and mediolateral (ML) direction to the pelvis.

**Methods:**

We developed an experimental setup to apply inertia in forward backward and or sideways directions. In two experiments nine healthy subjects walked on a treadmill at 1.5 km/h and 4.5 km/h with no load and with AP loads of 0.3, 1.55 and 3.5 kg to the left ankle in the first experiment and combinations of AP and ML loads on the pelvis (AP loads 0.7, 4.3 and 10.2 kg; ML loads 0.6, 2.3 and 5.3 kg). We recorded metabolic rate, EMG of major leg muscles, gait parameters and kinematics.

**Results & discussion:**

Adding 1.55 kg or more inertia to the ankle in AP direction increases the pelvis acceleration and decreases the foot acceleration in AP direction both at speeds of 4.5 km/h. Adding 3.5 kg of inertia to the ankle also increases the swing time as well as AP motions of the pelvis and head-arms-trunk (HAT) segment. Muscle activity remains largely unchanged.

Adding 10.2 kg of inertia to the pelvis in AP direction causes a significant decrease of the pelvis and HAT segment motions, particularly at high speeds. Also the sagittal back flexion increases. Lower values of AP inertia and ML inertias up to 5.3 kg had negligible effect.

In general the found effects are larger at high speeds.

**Conclusions:**

We found that inertia up to 2 kg at the ankle or 6 kg added to the pelvis induced significant changes, but since these changes were all within the normal inter subject variability we considered these changes as negligible for application as rehabilitation robotics and assistive devices.

## Background

In Robot aided gait training the trend is moving from position controlled robots as the early Lokomat [1] towards force controlled robots [2]. Where position controlled robots impose a gait pattern on a patient, force controlled robots offer the possibility to provide corrective- or guiding forces when needed. These “Assist As Needed” control algorithms [3-5] facilitate active participation, which have a positive effect on the rehabilitation process [6]. A prerequisite for assist as needed is that the robot does not affect gait when no assistance is needed, i.e., the robot must be able to minimize the interaction forces. This is known as “zero impedance control” [3] or transparent mode.

The target of transparent mode is to minimize interaction force between robot and subject; however zero interaction is impossible if the interaction force itself is the input for the control. The remaining impedance can be expressed in mechanical impedances such as inertia, damping, friction, stiffness, and combinations. Performance of gait training robots and other devices for force interactions with humans is often expressed in these mechanical impedances. Most impedances can be compensated for completely with control algorithms, such as admittance control [7]. Inertia, however usually cannot be compensated for completely, especially when passivity has to be guaranteed [8]. In robotic gait rehabilitation, this means that the inertia of the robot is perceived by the patient. Therefore it is important to know the effect of added inertia on gait, more specific, the effect of added inertia on legs and trunk.

Several studies have investigated the effect of mass added to body parts. These studies can be divided in two groups. The first group applied weights to body parts. These weights introduced inertia and a downward force due to gravity. In these studies it cannot be distinguished if the found effects are elicited by pure inertia or weight. The second group did compensate for the weight e.g. by means of body weight support, thus these studies assessed the effect of pure inertia. Adding inertia of 25-50% to the trunk of body mass resulted in an increase of energetics [9] and muscle activity [10]. Gait parameters remained unchanged [11] or change hardly (<3% [10]). The effect of inertia only on gait kinematics has not been assessed, however the effect of added weight has. The gravity component of the added weight has a significant effect on gait [9], therefore the found effects caused by added weight are likely to differ from effects caused by pure inertia. Table 1 summarizes the found effects of added inertia in previous studies.

**Table 1:** Effect of added inertia during walking

**Quantity**	**Effect of 25% body mass added to the trunk**	**Effect of mass added to the foot 4 kg**
Metabolic rate	+18% [9]	+36% @ 4 kg at foot [12]
		+8.2% @2.8 kg at proximal shank [13]
Muscle activity	+21% Soleus [10]	~0 [13]
Gait parameters	~0 [11] – 3% [10]	+2% swing time
		+1% Stride time
		@2.8 kg at proximal shank [13]
Gait kinematics	Unknown	Unknown

No literature was found on the effect of inertia added to the legs, only the effects of adding weight to legs have been assessed. Browning et al. showed that 4 kg attached to the ankle caused an increase of metabolic rate (36%) which is close to the effect of adding 16 kg added on the waist (32%) [12]. Royer and Martin [13] added weight in several distributions: the total weight remained constant (5.64 kg), while the moment of inertia with respect to the hip joint) varied. The largest increase was found in metabolic rate (+8.2%) when 2.82 kg was added to the proximal shank; other effects remained small. Though the weights added to the feet and legs are considerably less than the weights added to the pelvis by Grabowski [9], the effect of gravity on these added weights is expected to be considerable

To design robots for gait training, it is important to assess the effect of added inertia on gait, and ideally the inertia that the robot displays to the subject is so low that the effect on gait is negligible. Our objective is to establish this threshold for added inertia. The above-mentioned studies give an indication of this threshold, but there are some limitations. First, in all studies on ankle and leg loading, weight is added instead of inertia. Second, all studies that assessed the effect of added inertia on the trunk/pelvis did so by adding weights to a subject and compensating for the gravity of the weight by a body weight support system. A body weight support suspended on a fixed point has an equivalent of a stabilizing effect as a spring in a horizontal plane. Furthermore Aaslund and colleagues [14] have shown that the harness itself, without applying body weight support, has an effect on gait kinematics. Third, no study assessed the effect of inertia on gait kinematics. Fourth, in the different studies relatively large added inertias (~20 kg) were added on the trunk, whereas interaction control algorithms are expected to be able to reduce the displayed inertia to values below 10 kg [15,16]. The fifth limitation is that all studies applied equal inertia in all three translational degrees of freedom dimensions, while each controlled degree of freedom can be tuned independently, resulting in different inertias in different degrees of freedom. The last limitation is that the existing studies did not establish a threshold below which added inertia leaves gait unaffected, and thus below which a robot is transparent, and above which gait is affected.

The aim of this paper is to assess the effect of inertia added to the pelvis and the ankle. The first experiment adds inertia on the pelvis in anterior posterior (AP) and mediolateral (ML) direction; the second experiment adds inertia in AP direction on the ankle. We quantified the effect of inertia on gait parameters, gait kinematics, energetics, and muscle activity. These parameters are commonly used in gait analysis [9-11], and therefore are selected in our study, assuming that this set of parameters suffice in quantifying changes of gait. Moreover, a threshold is estimated for the allowable inertia of the gait training robot, below which walking in the gait training robot resembles normal walking. This serves as a recommendation for the design of transparent gait training robots.

We hypothesize that adding inertia elicits an increase of energetics or a decrease in gait motions (joint rotations, segment motions) or both. We base this on Newton’s second law:

F=ma

When inertia increases the first possible effect is that the subject exerts more force (muscle activity and energetics) to maintain the motion (acceleration). For extra inertia on the ankle, we expect an increase of muscle activity for push -off and stance preparation, since the acceleration and deceleration are highest in these phases. The second possible effect is that acceleration will decrease when a mass increases, while the subject maintains his effort (no change in energetics and muscle activity. For loads on the pelvis this implies smaller motions of the pelvis and trunk. For the ankle this would imply shorter stride length, but this inadvertently leads to an increase in cycle time (if speed is kept constant).

Finally we hypothesize that the effect of inertia is larger at high speeds, since in general accelerations of the segments are higher at high speeds, thus the absolute effect of a change in inertia is likely to elicit a larger absolute change in energetics.

## Method

### Subjects

Both experiments were executed with nine healthy subjects. All subjects signed an informed consent before the experiment. See Table 2 for subject data.

**Table 2:** Subjects data

	**Ankle experiment**	**Pelvis experiment**
Sex	7 men; 2 women	7men; 2 women
Weight	72.4 ± 12.5 kg	74.9 ± 9.0 kg
Height	1.81 ± 0.09 m	1.80 ±0.10 m
Age	25.1 ± 5.2 years	30.9 ± 10.3 years

### Apparatus

To add pure inertia, we designed a mechanism that connects the subject to two modules with adjustable inertias through a light-weight pelvis strap or ankle strap. The pelvis strap contains a light-weight bar, a rigid belt, and a trapezium construction, that allows pelvis rotation in the coronal plane.

A single module of adjustable inertia consists of a horizontal bar connected with spherical joints to a stand at one end and to the pelvis strap at the other end. Dumbbell weights are mounted on the bar. A steel wire is connected to the stand and the joint with the strap to assure vertical fixation of the bar, allowing only rotation of the bar and module around the vertical axis of the stand. The location of the dumbbell weight on the bar determines the added inertia to the segment, according to (1).

(1) $$M_{\mathrm{direction}}^{\mathrm{segment}}=\xi^{2}M^{\mathrm{dumbbell}}+M_{\mathrm{direction}}^{\mathrm{apparatus}}$$

$M_{\mathrm{direction}}^{\mathrm{segment}}$ denotes the added inertia on the segment in a specific direction; M^dumbbell^ is the mass of the dumbbell weights; $M_{\mathrm{direction}}^{\mathrm{apparatus}}$ is the inertia of the construction without the dumbbell weights at the segment (ankle 0.3 kg; pelvis anterior-posterior 0.58 kg; pelvis mediolateral 0.41 kg). Parameter ξ is the effective inertia gearing of the dumbbell weights, determined by the location of the dumbbell weight on its bar (see Table 3).

**Table 3:** Parameter values for pelvis AP and ML loading and ankle AP loading

**AP load on pelvis**			**ML load on pelvis**			**AP load on ankle**		
	**ξ**	$M_{\mathrm{AP}}^{\mathrm{pelvis}}$		**ξ**	$M_{\mathrm{ML}}^{\mathrm{pelvis}}$		**ξ**	$M_{\mathrm{AP}}^{\mathrm{foot}}$
1	0.10	0.7 kg	1	0.12	0.6 kg	1	0.0	0.3 kg
2	0.50	4.3 kg	2	0.36	2.3 kg	2	0.50	1.5 kg
3	0.80	10.2 kg	3	0.57	5.3 kg	3	0.80	3.5 kg

**Figure 1:**
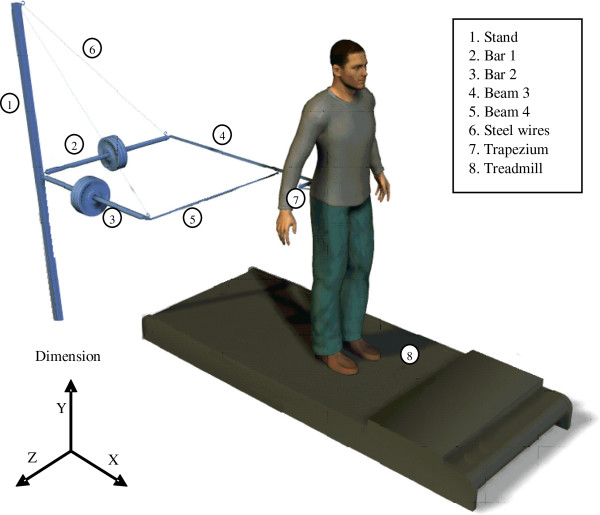
Apparatus for applying inertia to the pelvis in AP and ML direction independently.

**Figure 2:**
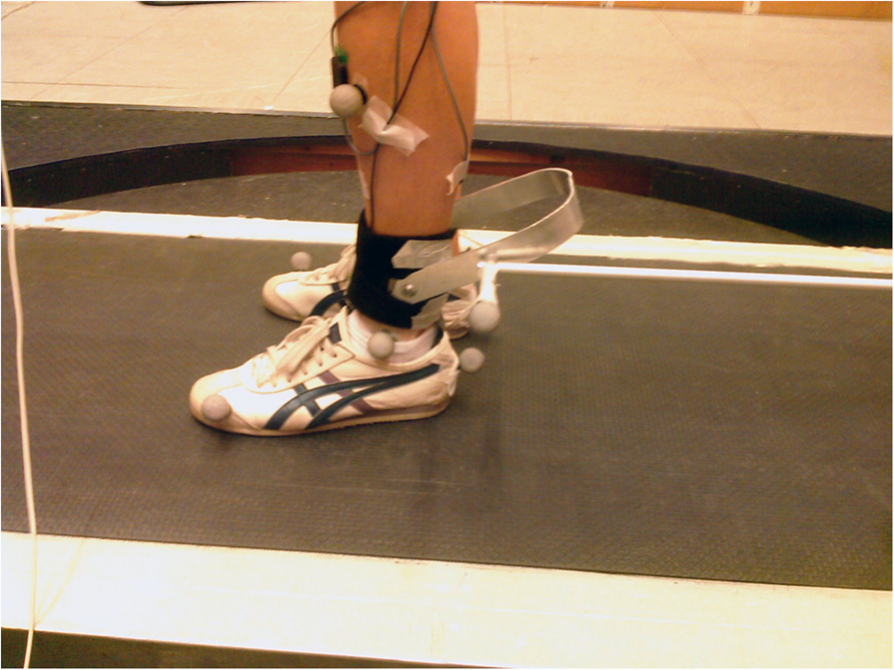
Apparatus attached to the ankle.

For the pelvis experiment we used two modules to apply AP load and ML load independently (see Figure 1 and Additional file 1); for the ankle experiment a single module was used to apply inertia in AP direction only (see Figure 2). In both studies, the inertia in other translations and rotations added by the apparatus is negligible.

We used loads that are similar to the loads used in the study of Browning and colleagues [12] (2 – 4 kg on the foot; 4 – 16 kg on the waist).

### Recordings

The effects of added inertia were assessed by quantifying kinematics, muscle activity and energetics.

### Kinematics and gait parameters

Motions were measured using an optical tracking system (Vicon Oxford Metrics, Oxford, UK). Twenty one reflective markers were attached to the human body; these markers were attached on both sides of the subject. Four markers were placed on the upper extremity (shoulders, front trunk and back trunk), five markers were placed on the pelvis (sacrum, left and right anterior superior iliac spine, left and right posterior superior iliac spine), on each leg seven markers were placed (femur, knee, tibia, malleolus, heel, fifth metatarsal joint). In both experiments two extra markers were placed on the apparatus, one on the stand and one on the strap near the ankle or pelvis. All markers were recorded at a sampling rate of 120 Hz by means of optical tracking.

### Muscle activity

The muscle activity was measured by recording the electromyography (EMG) from eight different muscles of the right leg: (1) the gluteus maximus, (2) gluteus medius, (3) rectus femoris, (4) vastus lateralis, (5) biceps femoris (6) gastrocnemicus medialis (7) soleus, and (8) tibialis anterior. The analogue signals were sampled at 1024 Hz and recorded with a Bagnoli system (Delsys, Boston, USA). Amplified EMG data was synchronized with the VICON System. Electrodes were placed over the muscle bellies according to the Seniam guidelines [17].

### Energetics

The energy expenditure was measured by the Oxycon Pro system (Jaeger, Hoechberg, Germany). Subjects were connected to the Oxycon with a flexible tube making an airtight seal to a facemask, measuring oxygen consumption (VO2) and volume expiration (VE). The heart rate of the subjects was measure at the index finger by a pulse-oximeter. Every five seconds (0.2 Hz) all parameters were measure and stored on the personal computer that was connected to the Oxycon.

### Experimental protocol

Both experiments started with two conditions in which subjects were walking on a treadmill at 1.5 km/h and 4.5 km/h without being attached to the system, called “no load” conditions (NL). These trials were followed by a randomized sequence of the added inertia- and speed conditions. In the pelvis experiment subjects walking with every combination of three loading conditions in AP and ML direction (see Table 3). In the ankle experiment, three loadings were applied in AP direction (see Table 3). Combining the loading conditions with speeds, resulted in 18 different loaded conditions for the pelvis-experiment and six loading conditions for the ankle-experiment. All trials consist of 3 minute walking.

### Data analysis

The last 12 VO2 samples (1 minute) were converted to VO2 rate per subject weight.

All kinematics and EMG data were split into individual stride cycles, determined by movement of the left heel marker [18]. Only the last 30 seconds of each trial was analyzed to eliminate the transition effects.

Marker data was converted to joint- and segment kinematics using custom written software, resulting in flexion and abduction of the left hip, left knee flexion, left plantar flexion, and back sagittal- and frontal rotation. For the pelvis and trunk the AP and ML motions and for the left foot the AP motion are analyzed in terms of position and acceleration. For the joint angles and segment motions, the range of motion (RoM) was calculated as the difference between the maximum and minimum value within a stride cycle.

We calculated the following gait parameters: cycle time [s], double stance time [s], swing time left [s], stance time left [s], step width [m] and stride length [m].

**Figure 3:**
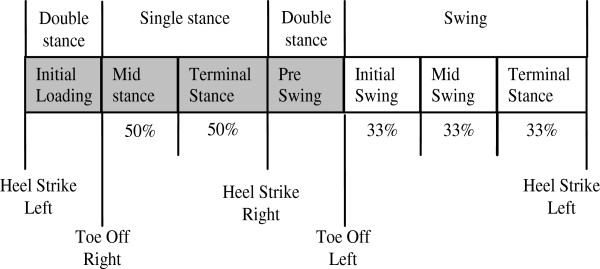
Phases of gait.

The raw EMG data was band passed filtered at 10–25 Hz with a second order (zero-lag) Butterworth filter. The filtered EMG data per subject per muscle was normalized to its maximal activity over the last 30 seconds. Mean muscle activity was calculated over seven intervals per step as described by Van Asseldonk [3]. The mean activity per interval was averaged per subject per trial. The double stance starting at left heel strike ending with toe off right defines *initial loading*; the stance phase is divided in two periods of equal length: *mid stance*, *terminal stance*, The double stance from heel strike right to toe off left defines the *pre swing;* the swing phase is divided in three periods of equal length: *initial swing*, *mid swing* and *terminal stance*. The intervals are described in Figure 3.

### Statistical analysis

The results of our research will be used as requirements gait training robots that are transparent. This implies walking in the gait training robot should resemble free walking. To quantify this resemblance we first checked the statistical significance first. However, this method does not take into account the variance within a subject (i.e. variance between steps). Even if differences are consistent, as they are significant, they may be small relative to the normal variance within a subject. Since our focus is to state requirements for gait training robots that allow normal walking and thus also the variability of normal walking, any effect that is smaller than the variability of walking is deemed ‘negligible’.

First we tested whether the NL conditions differed significantly from the BLSN condition to assess whether merely attaching the mechanical setup already affected the walking pattern. Subsequently we assessed the effects of the different loads.

To assess whether inertia had a significant effect on gait, we performed a two-way (velocity, AP load) repeated measures (ANOVA) in the ankle experiment; we used a three-way (velocity, AP load, ML load) in the pelvis experiment. We examined the main effects of load and the interaction effects between load and speed. Pair wise comparisons were performed on main- and interaction effects that are significant.

The intra subject variability (ISV) is calculated as twice the standard deviation in the baseline condition for a single subject. For data that is cut into steps (all except energetics) the standard deviation is taken over the number of steps. For the energetics the standard deviation is taken over the number of samples. If a parameter change due to added inertia does not exceed the averaged intra-subject variability the effect is judged as ‘negligible’.

(4) $$I\bar{S}V=2\frac{1}{\mathrm{nSubjects}}\sum_{i=1}^{\mathrm{nSubjects}}\sigma_{\mathrm{BSLN}}^{\mathrm{iSubject}}$$

In total we observed 31 parameters: energetics (1 parameter), gait parameters (6 parameters), joint angles (6 parameters), segment translation (5 parameters) and accelerations (5 parameters), and EMG activity (8 parameters). In both experiments 261 tests are performed: 31 parameters are observed + 56 muscle-phase combinations, resulting in 87 main effects, 174 interaction effects with speed (high and low speed).

## Results

### Baseline validation

#### Effect of apparatus on the ankle

When the apparatus was attached to the subject’s ankle with the inertia of the apparatus only (minimal added inertia), no significant increase is found in energetics and gait parameters relative to the free walking on the treadmill. Hip flexion range of motion however increased significantly (p = 0.009). The motions of the pelvis segment increased significantly in AP direction in position range of motion (RoM) (p = 0.008) and acceleration RoM (p = 0.006). The head-arm-trunk (HAT) position RoM increased significantly (p = 0.028). There is also a significant interaction effect with speed on the range of motion of the pelvis (p = 0.025) and HAT (p = 0.028). The post hoc tests showed the increase at low speed (see Table 4).

**Table 4:** List of significant effects of baseline validation

**Measure**	**Parameter**	**Speed**	**NL**	**BSLN**
**Energetics**	**-**		**-**	**-**
Gait parameters	-		-	-
Joint angles	LHip flexion RoM [deg]		33.2 ± 3.9	34.4 ± 3.7*
Segment motions	Pos pelvis AP RoM [mm]		45.0 ± 10.2	50.3 ± 6.4*
		1.5 km/h	50.1 ± 12.6	58.4 ± 7.8*
	Pos HAT AP RoM [mm]		34.3 ± 8.0	39.0 ± 6.9*
		1.5 km/h	38.4 ± 10.2	46.2 ± 11.3*
	Acc pelvis AP RoM [m/s2]		3.49 ± 0.67	3.71 ± 0.67*
EMG	Soleus		0.10 ± 0.02	0.11 ± 0.02*
	Soleus - pre swing		0.103 ± 0.036	0.122 ± 0.043*
	Soleus - inital swing		0.055 ± 0.021	0.066 ± 0.026*
	Soleus - mid swing		0.054 ± 0.019	0.069 ± 0.028*
	Gluteus maximus - mid stance	1.5 km/h	0.125 ± 0.050	0.114 ± 0.050*

* Significantly different from NL (p < 0.05).

Minimum added inertia is compared with no load; The table lists significant main effects (independent of speed) and significant interaction effects with speed, but only at speeds that were significant in pair wise comparisons.

The soleus shows a significant (p = 0.005) increase of mean activity due to the apparatus and moreover, the apparatus has a significant (p = 0.011) interaction effect with phases, and pair wise comparisons show significant increase in pre swing, initial swing and mid swing. The gluteus maximus and the gastrocnemicus medialis show a significant interaction effect (p = 0.041 and p = 0.037 respectively) on phase × speed × load. Pair wise comparison shows only a significant decrease (p = 0.028) of gluteus maximus in mid stance at slow speed.

None of the changes exceeded the intra subject variability.

### Effect of apparatus on the pelvis

When the apparatus was attached to the subject’s pelvis with the inertia of the apparatus only (minimal added inertia), no significant increase is found in energetics relative to the free walking on the treadmill. Of the gait parameters, only the stride length showed a significant increase of load (p = 0.024) and load × speed (p = 0.030) (see Table 5). The pair wise comparison showed a significant increase at slow speed.

**Table 5:** List of significant and appreciable effects of baseline validation

**Measure**	**Parameter**	**Speed**	**NL**	**BSLN**
**Energetics**	**-**		**-**	**-**
Gait parameters	Stride length [m]		0.67 ± 0.04	0.70 ± 0.05*
		1.5 km/h	0.52 ± 0.06	0.58 ± 0.08*
			(0.05)	
Joint angles	Left plantar flexion RoM [deg]		25.2 ± 3.5	28.0 ± 5.4*
	LHip flexion RoM [deg]	1.5 km/h	29.8 ± 2.5	31.7 ± 3.7
		4.5 km/h	42.0 ± 3.4+	42.4 ± 3.5
Segment Motions	Pos pelvis AP RoM [mm]	1.5 km/h	53.5 ± 10.0	63.5 ± 11.7*
	Acc pelvis ML RoM [m/s2]		1.50 ± 0.22	1.63 ± 0.29*
	Pos HAT AP RoM [mm]	1.5 km/h	41.3 ± 8.9	48.8 ± 9.3*
	Pos Left foot RoM [mm]	1.5 km/h	546.71 ± 77.23	612.50 ± 88.64*
			(41.23)	
EMG	Soleus		0.09 ± 0.02	0.09 ± 0.02*
	Biceps femoris – mid stance	4.5 km/h	0.14 ± 0.05	0.12 ± 0.05*

* Significantly different from NL (p < 0.05).

Bold: change larger than the average intra subject variability given in brackets.

Minimum added inertia is compared with no load; the table lists significant main effects (independent of speed) and significant interaction effects with speed, but only at speeds that were significant in pair wise comparisons.

Of the joint angles, plantar flexion range of motion increased significantly (p = 0.010). The hip flexion range of motion has a significant interaction effect with speed (p = 0.028), but no significance was found in the pair wise comparisons.

There was a significant interaction effect of load and speed on the pelvis AP position RoM (p = 0.002) and the head-arm-trunk (HAT) segment (p = 0.029). In both cases pair wise comparison showed an increase at slow speed. The pelvis acceleration range of motion increased significantly (p = 0.048) with 0.13 m/s^2^.

The EMG showed a significant (p = 0.047) effect on the soleus (from 0.087 to 0.091) and a significant (p = 0.042) interaction effect of phase, load and speed at the biceps femoris. The pair wise comparison showed a significant decrease of the biceps femoris in mid stance during fast walking.

Of all significant changes, only the stride length and foot RoM at low speed exceeded the averaged intra subject variability.

### Effect of inertia on the ankle in AP direction

Adding inertia in AP direction on the ankle causes a significant increase in metabolic rate (p = 0.001), and in interaction with speed (p = 0.006). The pair wise comparison revealed a significant increase at high speed only (see Table 6).

**Table 6:** Significant main effects and speed interaction effects of inertia added to the ankle

**Measure**		**Speed**	**0.3 kg**	**1.55 kg**	**3.50 kg**
Energetics	VO2 [ml/min/kg]		10.96 ± 1.92	11.58 ± 1.93	12.26 ± 1.96*+
		4.5 km/h	13.56 ± 2.40	14.46 ± 2.21	15.65 ± 2.45*+
Gait parameters	Double stance time [s]		0.32 ± 0.04	0.30 ± 0.04*	0.29 ± 0.04*
	Swing time left [s]		0.43 ± 0.02	0.47 ± 0.02*	0.50 ± 0.03*
			(0.07)		
		1.5 km/h	0.49 ± 0.05	0.54 ± 0.05*	0.60 ± 0.05*
			(0.1)		
		4.5 km/h	0.37 ± 0.01	0.39 ± 0.01*	0.41 ± 0.04*
			(0.04)		
	Stride length [m]		0.66 ± 0.05	0.66 ± 0.05	0.62 ± 0.04
Joint- & Segment angles	Back frontal flexion RoM [deg]	1.5 km/h	5.73 ± 1.91	5.69 ± 2.11	6.23 ± 2.37
	Back frontal flexion RoM [deg]	4.5 km/h	8.98 ± 1.61	9.48 ± 2.24	8.48 ± 1.91
	Back sagittal flexion RoM [deg]	1.5 km/h	2.83 ± 0.89	2.66 ± 0.97	2.76 ± 1.11
	Back sagittal flexion RoM [deg]	4.5 km/h	2.91 ± 0.53	3.29 ± 0.97	3.57 ± 0.96
	Knee flexion RoM [deg]		59.47 ± 3.82	58.82 ± 3.42	55.35 ± 3.48*+
	Plantar flexion RoM [deg]		26.70 ± 2.55	25.25 ± 4.00	23.90 ± 2.07*
Segment Motions	Pos pelvis AP RoM [mm]		50.31 ± 6.45	56.15 ± 9.10*	64.05 ± 14.12*
	Acc pelvis AP RoM [m/s2]		3.71 ± 0.67	4.01 ± 0.73*	4.27 ± 0.73*+
			(0.53)		
		4.5 km/h	4.72 ± 1.08	5.32 ± 1.10*	5.70 ± 1.04*+
			(0.59)		
	Pos HAT AP RoM [mm]		38.99 ± 6.89	41.59 ± 7.00	47.75 ± 11.84
	Acc HAT AP RoM [m/s2]		2.35 ± 0.35	2.52 ± 0.38	2.63 ± 0.42*
		4.5 km/h	3.08 ± 0.53	3.36 ± 0.58	3.47 ± 0.58*
			(0.37)		
	Pos HAT ML RoM [mm]		63.28 ± 15.93	68.45 ± 19.77	65.75 ± 14.79
	Pos Foot AP RoM [mm]		720.82 ± 56.69	712.15 ± 48.15	692.17 ± 51.53*
	AccLFoot AP RoM [m/s2]		41.07 ± 3.61	37.38 ± 3.10*	33.48 ± 3.31*+
			(3.99)		
		1.5 km/h	22.65 ± 2.14	21.18 ± 1.54	19.15 ± 3.15*
		4.5 km/h	59.49 ± 6.16	53.58 ± 5.05*	47.82 ± 3.89*+
			(4.33)		
EMG	Soleus – terminal stance		0.21 ± 0.07	0.20 ± 0.06	0.19 ± 0.08*
	tibialis anterior - inital swing		0.18 ± 0.03	0.18 ± 0.03	0.16 ± 0.03*+
	tibialis anterior – mid swing		0.15 ± 0.03 (0.02)	0.14 ± 0.03	0.12 ± 0.03*

* Significantly different from 0.3 kg (p < 0.05).

+ Significantly different from 1.55 kg (p < 0.05).

Bold: change larger than the average intra subject variability indicated between brackets at baseline.

Loaded conditions are compared to baseline condition. The table lists significant main effects (independent of speed) and significant interaction effects with speed, but only at speeds that are significant in pair wise comparisons.

In the muscle activity there is an interaction effect of AP inertia with phase on the gluteus medius (p = 0.045), the vastus lateralis (p < 0.001), the soleus (p = 0.020) and the tibialis anterior (p < 0.001), but pair wise comparisons revealed significant decrease only of soleus in terminal stance and the tibialis anterior in initial swing and mid swing.

The double stance time decreased significantly (p = 0.008), whereas the swing time increased (p < 0.001), and has an interaction effect with speed (p = 0.028). The stride length decreased significantly (p = 0.009), but pair wise comparison showed not significant changes.

Of the joint angles, we found significant decreases in the knee flexion (p = 0.001) and the plantar flexion (p = 0.010). Also significant interaction effects with speed appeared at both trunk frontal (p = 0.025) and sagittal rotation (p = 0.011), but the pair wise comparisons revealed no significant differences, also no clear trends are visible.

The pelvis and HAT segment motions AP increased significantly in position (pelvis p < 0.001; HAT p = 0.009) and acceleration (pelvis p < 0.001; HAT p = 0.001); for both segments the acceleration also has a significant interaction effect with speed (pelvis p = 0.001; HAT p = 0.042), which is significant only at high speed according to pair wise comparison.

The HAT segment position RoM in ML direction changed significantly (p = 0.035) due to load, but no consistent increase or decrease. Also pair wise comparison did not reveal significant differences.

Finally the left foot RoM decreased significantly in position RoM (p = 0.015) and acceleration RoM (p < 0.001). The acceleration has a significant interaction effect with speed (p < 0.001), at both speeds as revealed by pair wise comparison, but the decrease is considerably larger at high speeds (see Table 6).

**Figure 4:**
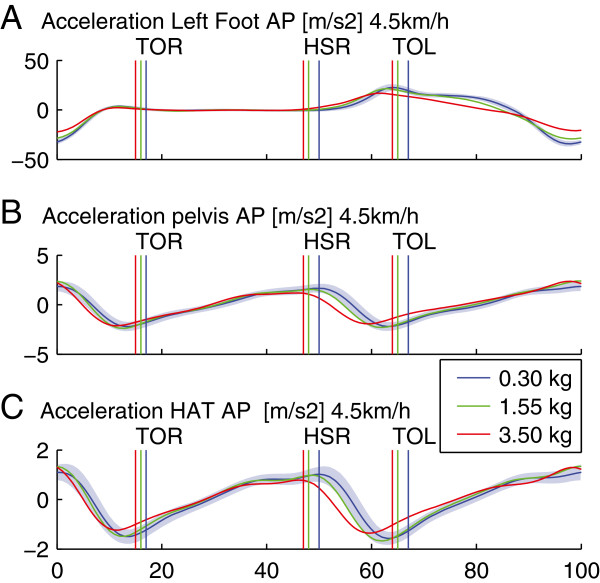
**Effect of inertia in AP direction on the ankle.** Acceleration profiles of the foot (**A**), pelvis (**B**) and trunk (**C**) as a function of the foot, pelvis and trunk as a function of the % gait cycle. Profiles are averaged across subjects and the shaded areas show the average intra subject variability. The cycle starts at 0% at heel strike left, followed by toe off right (TOR), heel strike right (HSR), toe off left (TOL) and ends with a heel strike left at 100%.

Of the significant changes due to adding 1.55 kg to the ankle, only two exceed the average intra-subject variability: at high speed the acceleration range of motion in AP direction of the pelvis and the left ankle. Adding 3.5 kg caused more changes that exceeded the ISV: the increase in swing time, the increase of pelvis- and HAT acceleration in AP direction, the decrease of the foot acceleration and the decrease of the tibialis anterior in initial swing. All these effects except for EMG are plotted in Figure 4.

### Effect of inertia on the Pelvis in AP direction

Adding inertia in AP direction on the pelvis during walking on a treadmill has no effect on energetics.

Of all the gait parameters only the stance time shows a significant (p = 0.050) interaction effect with speed, though a clear trend is not visible and also not revealed by pair wise comparisons (see Table 7).

**Table 7:** Significant main effects and speed interaction effects of inertia added to the pelvis in AP direction

**Measure**		**Speed**	**X 0.7 kg**	**X 4.3 kg**	**X 10.2 kg**
**Energetics**					
Gait parameters	Stance time left [s]	1.5 km/h	1.43 ± 0.17	1.40 ± 0.16	1.41 ± 0.16
		4.5 km/h	0.74 ± 0.04	0.74 ± 0.03	0.73 ± 0.03
Joint- & Segment angles	Hip abduction RoM [deg]		12.24 ± 2.59	11.84 ± 2.40*	11.32 ± 2.27*+
	Hip flexion RoM [deg]		37.04 ± 2.98	36.82 ± 2.54	36.14 ± 2.44*+
	Knee flexion RoM [deg]		61.35 ± 5.54	61.06 ± 4.86	59.93 ± 4.67
	Back sagittal flexion RoM [deg]		3.94 ± 0.96	4.54 ± 1.08*	5.29 ± 1.36*+
		1.5 km/h	4.25 ± 0.97	4.63 ± 0.99	4.98 ± 0.92*
		4.5 km/h	3.64 ± 1.16	4.45 ± 1.61*	5.60 ± 2.07*+
			(1.56)		
Segment Motions	Pos pelvis AP RoM [mm]		49.14 ± 6.05	46.75 ± 5.35	43.03 ± 5.29*+
	Acc pelvis AP RoM [m/s2]		3.88 ± 0.58	3.56 ± 0.41	3.06 ± 0.33*+
			(0.56)		
		4.5 km/h	5.04 ± 0.80	4.62 ± 0.62	3.85 ± 0.59*+
			(0.57)		
	Pos HAT AP RoM [mm]		37.37 ± 4.26	36.10 ± 4.89	33.89 ± 4.76*
	Acc HAT AP RoM [m/s2]		2.67 ± 0.29	2.55 ± 0.24	2.24 ± 0.14*+
			(0.36)		
		1.5 km/h	1.78 ± 0.28	1.66 ± 0.13	1.52 ± 0.13*+
		4.5 km/h	3.55 ± 0.38	3.45 ± 0.40	2.95 ± 0.19*+
			(0.35)		
EMG	Vastus lateralis - mid stance	1.5 km/h	0.23 ± 0.05	0.23 ± 0.05*	0.22 ± 0.06
	Vastus lateralis - terminal swing	4.5 km/h	0.06 ± 0.03	0.05 ± 0.02	0.06 ± 0.03*
	Gastrocnemius medialis - mid stance		0.13 ± 0.04	0.14 ± 0.04	0.14 ± 0.04*
	Soleus - mid stance		0.13 ± 0.04	0.14 ± 0.04*	0.14 ± 0.04*

* Significantly different from 0.3 kg (p < 0.05).

+ Significantly different from 1.55 kg (p < 0.05).

Bold: change larger than the average intra subject variability indicated between brackets at baseline.

Loaded conditions are compared to baseline condition. The table lists significant main effects (independent of speed) and significant interaction effects with speed, but only at speeds that are significant in pair wise comparisons.

The hip abduction, -flexion and knee flexion decrease at significantly (p = 0.002, p = 0.014 and p = 0.03) due to a load at the pelvis in AP direction. The trunk sagittal rotation increases significantly (p = 0.002). This has a significant interaction effect with speed as well (p = 0.003). Pair wise comparisons show that the increase is larger at high speed.

The pelvis and HAT motions in AP direction both decrease significantly in position RoM (pelvis p < 0.001; HAT: p = 0.003) and acceleration RoM (both p < 0.001). For both segments the acceleration RoM also has an interaction effect with speed (both p = 0.003). Pair wise comparison reveals that for the pelvis this occurs at high speed and high load only and for the HAT segment this occurs at both speeds, high load only, but the decrease is larger at high speeds (see Table 7).

The vastus lateralis has a significant interaction effect with speed and phase (p = 0.050). Pair wise comparison reveals a decrease during mid stance at slow speed, though this is not a clear consistent decrease (see Table 7). During terminal swing a decrease of activity is found at high speed. Both gastrocnemicus medialis and soleus show a significant interaction effect with phase (p = 0.018 and p = 0.022 respectively); both muscles showed an increase during mid stance.

**Figure 5:**
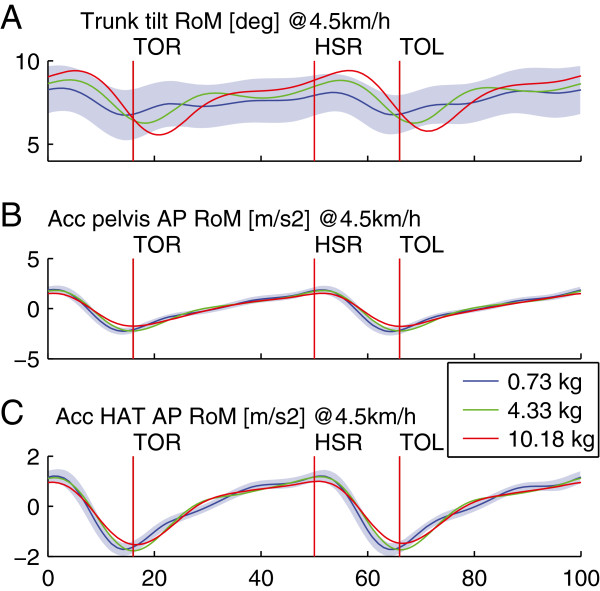
**Effect of inertia in AP direction on the pelvis.** Trunk tilt (**A**), pelvis mediolateral acceleration (**B**) and trunk mediolateral acceleration (**C**) as a function of the% gait cycle. Profiles are averaged across subjects and the shaded areas show the average intra subject variability. The cycle starts at 0% at heel strike left, followed by toe off right (TOR), heel strike right (HSR), toe off left (TOL) and ends with a heel strike left at 100%.

In short, although adding 4.3 kg to the pelvis in AP direction caused significant effects, none of these effects exceeded the averaged ISV. Adding 10.2 kg however did cause changes larger than the ISV: the increase of the trunk sagittal rotation, the acceleration in AP direction of pelvis and HAT segments (see Figure 5).

### Effect of inertia on the pelvis in ML direction

Adding inertia to the pelvis in ML direction during walking has no effect on energetics, gait parameters and joint angles. Of the segment motions only the acceleration range of motion of the pelvis and HAT segment in ML direction decreased significantly (p = 0.037 and p = 0.007), though pair wise comparisons revealed no significant changes.

The overall activity of the tibialis anterior has a significant change (p = 0.013), though a clear trend is not visible (see Table 8). And the vastus lateralis has a significant interaction effect with phase and speed (p = 0.004), but pair wise comparisons did not reveal significant changes.

**Table 8:** Significant main effects and speed interaction effects of inertia added to the pelvis in ML direction

	**Z 0.6 kg**	**Z 2.3 kg**	**Z 5.3 kg**
Acc pelvis ML RoM [m/s2]	1.61 ± 0.22	1.57 ± 0.24	1.54 ± 0.20
Acc HAT ML RoM [m/s2]	1.39 ± 0.23	1.37 ± 0.23	1.32 ± 0.20
Tibialis anterior	0.12 ± 0.02	0.11 ± 0.02	0.12 ± 0.02*

* Significantly different from 0.3 kg (p < 0.05).

None of the significant changes exceeded the average intra-subject variability.

## Discussion

We assessed the effect of adding inertia during walking on a treadmill in order to assess the effect of a gait training robot on gait. This study is novel in that we decoupled the inertia from gravitational effect and that we decoupled the inertia in different directions. We conducted two experiments, one with adding inertia to the ankle in anterior-posterior direction and one with adding inertia to the pelvis in AP and ML direction.

### Effect of the apparatus

In both experiments we first assessed the effect of the apparatus with minimum added inertia with free walking. The overall effect of the apparatus is negligible in both experiments. There were some significant changes, but these were very relatively small (few percent) and few in number: 11 for the ankle experiment and 10 for the pelvis experiment.

Most of these significant changes involve slow walking. All the loaded conditions, including the baseline conditions were randomized, but the free walking conditions were not randomized. Every subject started with slow walking. It is likely that the subjects were not completely familiarized with walking on the treadmill during the first trial i.e. free walking at slow speed. This could account for the significant interaction effects (load × speed) that were found in the baseline validation.

### Effect of AP load on ankle

Adding inertia to the ankle during walking on a treadmill caused several significant changes. We hypothesized an increase in effort by the subject in order to maintain gait patterns. We did see a significant increase in energetics; this was not accompanied by an increase in muscle activity of the left (loaded) leg. This can be explained by the fact that muscle activity of the right leg increased, however this was not investigated. An argument in favor of this explanation could lie in the fact that the pelvis and hat segment show increased acceleration mainly during the terminal swing phase of the left leg. Since the left leg is swinging, effort for increased acceleration is likely to be caused by the right leg.

Adding 1.55 kg inertia to the ankle caused an increased in metabolic rate of 5.7%, which is a little less than the 7.6% that Royer and Martin found when applying a weight of 1.2 kg to the ankle and 0.8 kg to the knee [13]. They also found a significant increase of the soleus activity, where we found a small decrease. In our study the added load did not require vertical acceleration during push-off, whereas the extra weight in their study did require extra effort in vertical acceleration.

The muscle activity of the loaded leg remained unchanged largely, except for a few decreases: The first is that of the soleus in terminal stance, which indicates a reduced effort in push off, which is also visible in the reduced plantar flexion range of motion. Consequently the foot acceleration decreases and the stride length decreases. Contrary to our hypothesis the subject reduces the effort to accelerate the foot and its extra inertia, and ‘accepts’ changed gait patterns. The second reduction of muscle activity is the tibialis anterior in swing phase, indicating a reduced effort to lift the toe, which is explained by a reduced push off: if the plantar flexion is reduced, less effort is needed to lift the toes for sufficient ground clearance. Another consequence of the decreased acceleration is the significant increase in the swing time for the left leg: it takes longer before the foot touches the ground. Also this change is larger than the ISV, and therefore stated as ‘appreciable’.

Adding 1.55 kg caused only an appreciable change in acceleration range of motion of the pelvis and left foot at high speeds only; the other 29 parameters remained unaffected. In both cases the changes just exceed the average intra subject variability. Therefore we conclude that walking with 1.55 kg added to the ankle in AP direction resembles normal walking.

Adding 3.5 kg also caused appreciable changes at low speeds and especially the changes of the pelvis and foot acceleration RoM at high speeds are much larger than the average intra subject variability. Therefore we conclude that walking with 3.5 kg added to the ankle in AP direction does not resemble normal walking.

### Effect of AP load on pelvis

We assessed the effect of adding inertia to the pelvis in AP direction during walking on a treadmill. We hypothesized that the effort remains unchanged and this is confirmed by the fact that energetics remain unchanged. The EMG activity does show significant changes, but, though significant, the changes are very small, and do not exceed the average intra subject variability.

As hypothesized the pelvis motions decrease due to added inertia in AP direction. Though the decreases are less than the average intra subject variability, the pelvis position- and acceleration RoM decrease significantly. As the trunk is connected to pelvis also the trunk motions decrease significantly. However the sagittal rotation between the trunk and the pelvis, the back sagittal flexion increases. A possible explanation is that the pelvis shows more sagittal rotation, this however was not investigated in the study. The inertial forces due to the added inertia may elicit a moment in the sagittal plane, causing the increased rotation.

Of all significant changes only the AP acceleration of the pelvis and hat segment and the sagittal rotation of the back exceed the average intra subject variability, only when 10.2 kg was attached. Therefore we consider walking with 4.3 kg to be similar to normal walking, whereas 10.2 kg does not resemble normal walking.

### Effect of ML load on pelvis

We assessed the effect of adding inertia to the pelvis in mediolateral direction during walking on a treadmill. We hypothesized that the ML motions of the pelvis would decrease, and correspondingly adding inertia did decrease the range of motion of the pelvis and HAT segment in ML direction significantly. These changes however are small and did not prove to be significant in pair wise comparisons. Therefore we conclude that 5.3 kg can be added on the pelvis in ML direction without affecting the gait.

### Weber fractions

During the experiments subjects claimed that they did perceive the inertia, in several condition of low and high inertia. This can be explained by the Weber fraction [19]. The smallest noticeable difference in weight (the least difference that the test person can still perceive as a difference), is proportional to the starting value of the weight Based on this, one could estimate what would be the just noticeable difference (JND) of added inertia, simply by taking the Weber fraction of the mass of the leg. For mass the Weber fraction is 1/10 [19,20] Applying this to the mass distribution of the human body gives a JND of 3.2 kg for the trunk and 0.15 kg for the foot (see Table 9). In our study the applied inertia was more than the JND except for the baseline and the pelvis ML 2.3 kg condition. This can explain why subject did perceive the difference even if physical measurement did not.

**Table 9:** Just noticeable difference for body segments according to the Weber fraction

	**Weight percentage**	**Weight [kg] (75 kg bodyweight)**	**JND [kg] according to Weber fraction (1/10)**
Trunk	43%	32.25	3.225
Upper leg	12%	9	0.9
Lower leg	5%	3.75	0.375
Foot	2%	1.5	0.15

### Comparisons between experiments

In our experiments we found that adding inertia to the ankle causes more effect that adding the same inertia to the pelvis. Browning found similar results [12], with added weights, meaning that the found effects cannot be ascribed to the gravitational component only. An explanation is given by the fact that the acceleration of the foot is ten times larger than the acceleration of the pelvis in AP direction. Similarly, the acceleration of the pelvis in forward direction is three times higher than the acceleration of the pelvis in ML direction.

As hypothesized the effects are larger at high speeds. In the ankle experiment four appreciable effects at high speeds are found, only one for low speed. In the pelvis experiment for AP loading, three appreciable effects at high speeds are found, none for low speed.

### Comparison with other studies

In the pelvis experiment we applied AP inertia up to 10.2 kg and ML inertia of 5.3 kg which is approximately 13% and 7% of the body mass. Grabowski applied inertia of 25% of body weight in all directions and found an increase in metabolic rate (+25%). In our study the metabolic rate remains unchanged when applying inertias to the pelvis in the horizontal plane only. McGowan and colleagues applied inertias equal to 25% and 50% of the body mass and found an increase of soleus activity at the late stance of 17% and 43%. They found that the soleus is the primary contributor to forward trunk propulsion [10] and that the soleus and gastrocnemicus both contribute in both support. In our study we reported no change of muscle activity in terminal stance, when adding 10.2 kg in AP direction or 5.2 kg in ML direction. However we did see a significant increase of both soleus and gastrocnemicus in mid stance. McGowan applied inertia in all directions including vertical hence affecting the AP component of propulsion and the vertical component of propulsion, whereas we applied inertia only in one direction, only affecting the horizontal component of propulsion. Comparing our results with found results from Grabowski and McGowan suggests that vertical motion of the pelvis may be the degree of freedom that is most sensitive to added inertia.

To find requirements for gait training robots, we started with applying inertia on one segment in one or two directions. Since nearly all robotic gait trainers have an interface to the lower shank and the pelvis, we applied inertia to the ankle and the pelvis. To obtain a complete set of requirements for gait training robots, the effect of inertia added to the knee should be investigated, as well as the effect of combined inertia’s added to the ankle, knee and pelvis.

## Conclusion

In order to allow normal walking in a gait training robot, the robot should be transparent. In our study we quantified the requirements for transparent walking. We assume that when energetics, kinematics and gait parameters are unaffected, transparency in gait training is guaranteed. We found that inertia up to 2 kg to the ankle or 6 kg added to the pelvis have negligible effect on energetics, kinematics and gait parameters. Therefore, for gait training robots to be transparent, they should display inertias less than the found thresholds.

## Competing interests

Jos Meuleman works at Moog, working on the design of a gait training robot.

## Authors’ contributions

JM conducted the research, data analysis and writing. EA and HK contributed in the design of the study and writing. All authors read and approved the final manuscript.

## Supplementary Material

Additional file 1 Movie of adding inertia to the pelvis in anterior-posterior and lateral direction independently. Click here for file

## References

[B1] Colombo G Joerg M Schreier R Dietz V Treadmill training of paraplegic patients using a robotic orthosis J Rehabil Res Dev 2000 37 693 700 11321005

[B2] Veneman JF Kruidhof R Hekman EE Ekkelenkamp R Van Asseldonk EH van der Kooij H Design and evaluation of the LOPES exoskeleton robot for interactive gait rehabilitation IEEE Trans Neural Syst Rehabil Eng 2007 15 379 386 17894270 10.1109/tnsre.2007.903919

[B3] van Asseldonk EHF Veneman JF Ekkelenkamp R Buurke JH van der Helm FCT van der Kooij H The Effects on Kinematics and Muscle Activity of Walking in a Robotic Gait Trainer During Zero-Force Control Neural Syst Rehabil Eng IEEE Trans 2008 16 360 370 10.1109/TNSRE.2008.925074 18713676

[B4] Emken J Harkema S Beres-Jones J Ferreira C Reinkensmeyer D Feasibility of manual teach-and-replay and continuous impedance shaping for robotic locomotor training following spinal cord injury IEEE Trans Biomed Eng 2008 55 322 334 18232376 10.1109/TBME.2007.910683

[B5] Emken J Reinkensmeyer D Robot-enhanced motor learning: accelerating internal model formation during locomotion by transient dynamic amplification IEEE Trans Neural Syst Rehabil Eng 2005 13 33 10.1109/TNSRE.2004.843173 15813404

[B6] Kwakkel G Kollen BJ Krebs HI Effects of robot-assisted therapy on upper limb recovery after stroke: a systematic review Neurorehabil Neural Repair 2008 22 111 121 17876068 10.1177/1545968307305457 PMC2730506

[B7] Linde RQ Lammertse P Haptic Master - a generic force controlled robot for human interaction Ind Robot Int J 2003 30 515 524 10.1108/01439910310506783

[B8] Buerger SP Hogan N Complementary Stability and Loop Shaping for Improved Human - Robot Interaction 23 IEEE Transactions on Robotics 232–244 (2007). doi:10.1109/TRO.2007.892229

[B9] Grabowski A Farley CT Kram R Independent metabolic costs of supporting body weight and accelerating body mass during walking J Appl Physiol 2005 98 579 583 15649878 10.1152/japplphysiol.00734.2004

[B10] McGowan CP Neptune RR Kram R Independent effects of weight and mass on plantar flexor activity during walking: implications for their contributions to body support and forward propulsion J Appl Physiol 2008 105 486 494 10.1152/japplphysiol.90448.2008 18556431 PMC2519947

[B11] De Witt JK Hagan RD Cromwell RL The effect of increasing inertia upon vertical ground reaction forces and temporal kinematics during locomotion J Exp Biol 2008 211 1087 1092 10.1242/jeb.012443 18344482

[B12] Browning RC Modica JR Kram R Goswami A The effects of adding mass to the legs on the energetics and biomechanics of walking Med Sci Sports Exerc 2007 39 515 525 10.1249/mss.0b013e31802b3562 17473778

[B13] Royer TD Martin PE Manipulations of leg mass and moment of inertia: effects on energy cost of walking Med Sci Sports Exerc 2005 37 649 656 10.1249/01.MSS.0000159007.56083.96 15809565

[B14] Aaslund MK Moe-Nilssen R Treadmill walking with body weight support: Effect of treadmill, harness and body weight support systems Gait posture 2008 28 303 308 10.1016/j.gaitpost.2008.01.011 18343664

[B15] Adams RJ Hannaford B **Control law design for haptic interfaces to virtual reality** Book Control law design for haptic interfaces to virtual reality 2002 (Editor ed.^eds.). City

[B16] Furuta K Kosuge K Shiote Y Hatano H Master–slave manipulator based on virtual internal model following control concept Robotics and Automation Proceedings 1987 IEEE International Conference 1987 567 572 *on*; *Mar 1987*23637990

[B17] Hermens H Freriks B Merletti R Stegeman D Blok J Rau G Disselhorst-Klug C Hägg G European Recommendations for Surface ElectroMyoGraphy 1999

[B18] Zeni JA Jr Richards JG Higginson JS Two simple methods for determining gait events during treadmill and overground walking using kinematic data Gait Posture 2008 27 710 714 10.1016/j.gaitpost.2007.07.007 17723303 PMC2384115

[B19] Ross HE Brodie EE Weber fractions for weight and mass as a function of stimulus intensity Quarterly J Exp Psychology Sect Human Exp Psychology 1987 39 77 88 10.1080/02724988743000042 3615942

[B20] Ross HE Brodie EE Benson AJ Mass-discrimination in weightlessness and readaptation to earth's gravity Expe Brain Res 1986 64 358 366 10.1007/BF00237752 3803477

